# Exercise and colorectal cancer survival: an updated systematic review and meta-analysis

**DOI:** 10.1007/s00384-022-04224-5

**Published:** 2022-07-27

**Authors:** Kay T. Choy, Kenneth Lam, Joseph C. Kong

**Affiliations:** 1 Department of Surgery, Austin Hospital, Melbourne, VIC Australia; 2 Department of Surgery, St. Vincent’s Hospital, Melbourne, VIC Australia; 3 Division of Cancer Surgery, Peter MacCallum Cancer Centre, Melbourne, VIC Australia; 4 Division of Cancer Research, Peter MacCallum Cancer Centre, Melbourne, VIC Australia; 5 Sir Peter MacCallum Department of Oncology, University of Melbourne, Parkville, VIC Australia

**Keywords:** Exercise, Colorectal cancer survival

## Abstract

**Purpose:**

The benefit of exercise to colorectal cancer patients has been advocated. However, comparative data to quantify the survival benefit is lacking. The aim of this review was to assess the effect of exercise on colorectal cancer survival.

**Methods:**

An up-to-date systematic review was performed on the available literature between 2000 and 2021 on PubMed, EMBASE, Medline, and Cochrane Library databases. All studies reporting on the impact of exercise and colorectal cancer outcomes in patients treated for non-metastatic colorectal cancer were analysed. The main outcome measures were the overall survival (OS), cancer specific survival (CSS) and disease free survival (DFS).

**Results:**

A total of 13 prospective observational studies were included, accounting for 19,135 patients. Compared to negligible physical activity, overall survival (OS) was significantly increased for both moderate and highest activity group (HR 0.82, 95% CI: 0.74–0.90, *p* < 0.001 and HR 0.64, 0.56–0.72, *p* < 0.001 respectively). This was also reflected in cancer specific survival (CSS) analysis, but not disease-free survival (DFS) analysis.

**Conclusion:**

Exercise was associated with an increased in overall survival after a colorectal cancer resection. This would support the promotion of exercise interventions amongst colorectal cancer patients.

## Introduction

Increasing evidence shows physical activity to be associated with improved colorectal cancer (CRC) prognosis [1]. Most of the available studies to date have investigated post-diagnosis physical activity in association with prognosis after CRC diagnosis. This has shaped current guidelines that recommend cancer survivors to avoid inactivity and to perform ≥ 150 min of moderate or ≥ 75 min of vigorous activity per week or an adequate combination of both [2, 3].

However, these studies vary in their reported outcomes with conclusions on CRC-specific survival (CSS), disease-free survival (DFS) and OS often interchanged with one another [4, 5], leading to much confusion. This has led to calls for large-scale prospective patient cohorts to comprehensively ascertain the effect of physical activity while considering each specific outcome measure [1].

Therefore, the aim of our study was to provide an up-to-date review of evidence to elucidate the association between physical activity and CRC prognosis after a curative resection. While previous systematic reviews have established a favourable OS with exercise, our study included sub-analysis of CSS as a “net” measure after removing competing causes of death and DFS, which describes the period after treatment during which there are no signs and symptoms of the colorectal cancer that was treated [6, 7].

## Methods

### Search strategy

All relevant published studies were identified through a computer-assisted search of PubMed, EMBASE, Medline, and Cochrane Library databases between the years of 2000 and 2021. The following medical subject heading (MeSH) terms and text words were used for the search in all possible combinations: “exercise” AND “colorectal cancer” OR “colorectal cancer survival.” The cited references in each retrieved paper were also checked to ensure that all publications relevant to this study were captured. The last search date for this study was 31 December 2021.

### Selection of studies

This study was conducted in accordance to the preferred reporting items for systematic reviews and meta-analyses (PRISMA) guidelines [8]. All article titles and abstracts were screened firstly, with all potentially relevant studies then subsequently retrieved for full-text review. Article selection of articles was based on the following inclusion criteria: adult population, after curative resection (R0) of non-metastatic CRC in order to identify all studies comparing exercise and colorectal cancer survival. All non-English studies, letters, perspectives, and conference abstracts were excluded.

### Definitions

The quantification of exercise activity differed slightly between all the papers, but most studies utilized the metabolic equivalent task (MET) score. One MET is the energy expenditure for sitting quietly, also referred to as the resting metabolic rate. MET scores are therefore defined as the ratio of the metabolic rate associated with specific activities divided by this resting metabolic rate. The values from the individual activities were summed for a total MET-hours per week score. Categories of MET-hours per week were predefined as 3 or less, 3.1 to 9, 9.1 to 18, 18.1 to 27 or greater than 27, to correspond to the equivalent of less than 1, 1 to less than 3, 3 to less than 6, 6 or more hours per week of walking at an average pace, consistent with prior analysis [6].

Amongst our analysed studies, some also reported on pre and post diagnosis activity. This was defined by Meyerhardt et al. (2006) with pre-diagnosis being 6 months prior to diagnosis whereas post-diagnosis included the period 1–4 years after diagnosis for the length of follow-up [9].

Furthermore, the lifetime average leisure time was used to retrospectively quantify for each 10-year age from 20 to 80 years. Patients provided information retrospectively on their task-specific MET-h/week scores for each recorded decade. Using this information from all ages, the activity-specific lifetime average MET-H/week score was calculated considering the current age of the patient and the years spent in each decade. This allowed classification of pre-diagnosis exercise level of the immediate 10 years prior, as well as post-diagnosis level looking at exercise within 12 months of diagnosis.

As the main aim of the study was to quantify the survival benefits of exercise in colorectal cancer, the various categories of physical activity were standardized. Irrespective of the measure of calculation, the categories of exercise were broken down into nil/minimal, moderate level 1, moderate level 2 and high intensity. This would allow the greatest number of included studies for comparison, while attempting to demonstrate any possible dose related changes in survival outcomes.

The main outcome measures were OS, and secondary measures looking at CSS as well as DFS were also calculated.

### Data extraction

Two reviewers (KTC and KL) independently extracted the data from the included studies using a standard data extraction form. Any discrepancies were resolved by consensus between the two reviewers and the supervising author (JCK).

### Statistical analysis

For each outcome measure, the hazard ratio (HR) with its associated 95% confidence interval (CI) was collected, with the comparison denominator as nil or minimal exercise (*HR* = 1). A pooled HR was performed using the random effect model due to heterogeneity. *I* [2] statistics were performed to assess for inter-study heterogeneity and the Newcastle–Ottawa scale (for non-randomized studies) was used to assess the quality of each non-randomized study. A *p*-value of < 0.05 was considered significant. All data analysis was performed in RStudio Team (2015).

## Results

### Search results and included studies

There were 112 citations identified from the initial search. Six additional studies were included from references of identified articles. After screening for full text reviews, a total of 13 studies were included in the study.

Three studies reported physical activity as a dichotomy — no activity versus activity (usually more than 1 h per week) [7, 10, 11]. Out of the remaining ten studies, two studies described activity in terms of negligible, insufficient, and sufficient discrete variables [12, 13]. The remaining eight studies calculating MET-hours per week — predefined as 3 or less, 3.1 to 9, 9.1 to 18, 18.1 to 27 or greater than 27, to correspond to the equivalent of less than 1, 1 to less than 3, 3 to less than 6, 6 or more hours per week of walking at an average pace — was consistent with prior analyses [1, 2, 6, 14–19].

Finally, out of these 13 studies, five looked at post-diagnosis activity levels [6, 10, 11, 15, 18], two looked at pre-diagnosis levels [7, 13] while the remaining six looked at both pre and post diagnosis activity levels [1, 6, 12, 14, 16, 17, 19].

### Study design and quality

All 13 studies were non-randomized two prospective observational studies. These non-randomized studies scored 6 or more on the Newcastle–Ottawa Scale.

### Patient characteristics

The median age for patients included in this study ranged between 57.9 and 72 years. While two out of the 13 studies only included female patients with one other study looking at male patients alone, the other 10 studies had a balanced distribution between sexes (Table 1). In terms of disease characteristics, there was a relatively similar distribution of colonic versus rectal primary tumours (Table 1).

**Table 1:** Study and patient characteristics of all studies

Author (year)	Definition of exercise	Pre/post-diagnosis	No. of patients	Age	Male	Location of tumour
Phipps et al. (2018) [10]	< 1 episode of vigorous activity per month, > 1 episode of vigorous activity per month	Post-diagnosis	487, 1505	57.8	222, 818	47% right, 53% left
Walter et al. (2017) [1]	MET-H/week (0–25.4, 25.4–43.5, 43.5–65.4, > 65.4) (lifetime average leisure time physical activity)	Pre-diagnosis	774, 768, 769, 769	70, 70, 69, 68	471, 419, 464, 483	59.4% colon, 40.6% rectum
	MET-H/week (0–25.4, 25.4–43.5, 43.5–65.4, > 65.4) (latest average leisure time physical activity)	Post-diagnosis	786, 750, 762, 766	72, 70, 68, 67	464, 415, 457, 489	
Park et al. (2017) [11]	< 1 h/week, > 1 h/week	Post-diagnosis	97, 203	61.6, 60.6	46, 112	56.7% colon, 43.3% rectum
Tamakoshi et al. (2017) [13]	No habit, 1–2 times/week, > 3 times/week	Pre-diagnosis	1359, 352	-	-	61% colon, 39% rectum
Arem et al. (2015) [14]	Never, < 1 h/week, 1–3 h/week, 4–7 h/week, > 7 h/week (prediagnosis LTPA)	Pre- and post-diagnosis	619, 418, 994, 896, 870	64.3, 64.4, 64.4, 64.7, 64.9	68, 66, 65.9, 65.6, 66	Not reported
Jeon et al. (2013) [15]	MET-h/week (< 3, 3–17.9, > 18)	Post-diagnosis	81, 96, 60	63, 60, 59.5	38, 59, 43	Not reported
Campbell et al. (2013) [16]	MET-h/week (< 3.5, 3.5–8.74, > 8.75)	Pre- and post-diagnosis	255, 943, 1064	-	165, 487, 619	73.4% colon, 26.6% rectum
Kuiper et al. (2012) [17]	MET-h/week (0, 0–2.9, 3.0–8.9, 9.0–17.9, > 18)	Pre- and post-diagnosis	234, 166, 350, 312, 277	65.6, 65.1, 65.7, 65.9, 66.3	All women	80.8% colon, 19.2% rectum
Baade et al. (2011) [12]	Sedentary, insufficient active pa/week, sufficient active pa/week	Pre- and post-diagnosis	748, 484, 593	-	289, 126, 208	63.7% colon, 36.3% rectum
Meyerhardt et al. (2009) [18]	MET-h/week (< 3, 3.1–9, 9.1–18, 18–27, > 27)	Post-diagnosis	102, 125, 101, 81, 252	72, 69, 68,68, 69	All men	Not reported
Meyerhardt et al. (2006) [6]	Pre-diagnosis Met-h/week (< 3, 3.1–9, 9.1–18, 18–27, > 27)	Pre- and post-diagnosis	142, 152, 118, 161	65, 65, 63, 66	All women	81% colon, 19% rectum
	Post-diagnosis Met-h/week (< 3, 3.1–9, 9.1–18, 18–27, > 27)		167, 146, 97, 144	65, 65, 64, 64	All women	80% colon, 20% rectum
Haydon et al. (2006) [7]	Non-exercises, exercises	Pre-diagnosis	297, 229	67.6, 68.6	51, 52	66.5% colon, 33.5% rectum
Meyerhardt et al. (2006) (6)	MET-h/week (< 3, 3–8.9, 9–17.9, 18–26.9, > 27)	Post-diagnosis	273, 187, 137, 84, 151	61, 61, 59, 59, 59	44, 57, 64, 67, 66%	Not reported

### Meta-analysis of surgical outcomes

#### Overall survival

Compared to negligible activity, physical activity was associated with an 18 to 36% reduction in the overall mortality. Overall, the overall survival yielded a hazards ratio of 0.82 for the moderate activity 1 group (HR 0.82, 95% CI: 0.74–0.90, *p* < 0.001) with that increasing to 0.64 (0.56–0.72, *p* < 0.001) for the highest activity group (Table 2).

**Table 2:** Survival analysis per level of exercise/physical activity

	**Moderate 1**	**Moderate 2**	**Highest**
Overall survival (OS)	0.82 (0.74, 0.90), *p* < 0.001	0.66 (0.54, 0.81) *p* < 0.001	0.64 (0.56, 0.72), *p* < 0.001
Cancer-specific survival (CSS)	0.88 (0.77, 1.01), *p* = 0.078	0.66 (0.55, 0.78), *p* < 0.001	0.69 (0.57, 0.84), *p* < 0.001
Disease-free survival (DFS)	0.92 (0.79, 1.08), *p* = 0.327	0.85 (0.58, 1.23), *p* = 0.391	0.85 (0.71, 1.02), *p* = 0.072

#### Cancer-Specific survival

Cancer specific survival analysis yielded similar results. Moderate activity 1 group was nearly significant with the upper limit of 95% confidence interval crossing the 1.0 null effect line (HR 0.88, 95% CI 0.77–1.01, *p* = 0.078). However, moderate activity 2 and highest activity group showed a significant increase in cancer specific survival of 31–34% (*p* < 0.001).

#### Disease-free survival

**Fig. 1:**
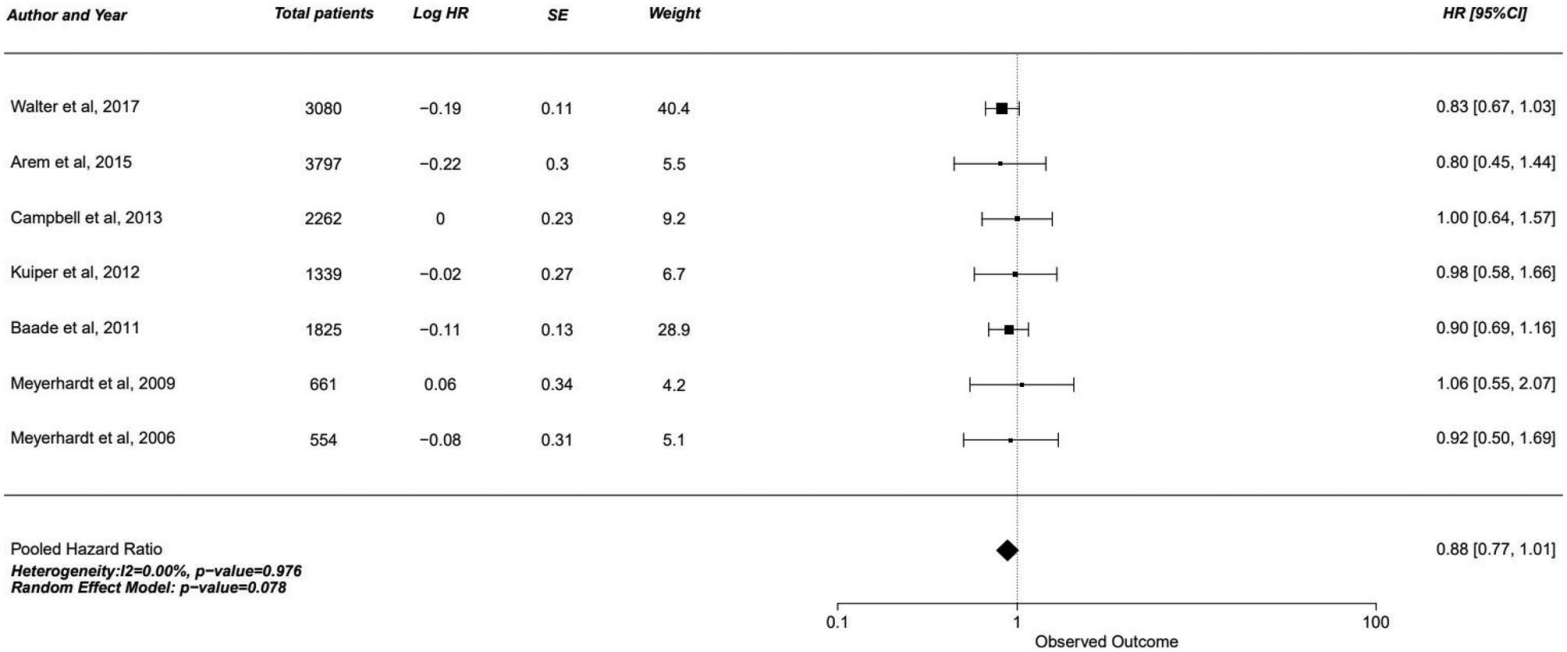
Forest plot of studies comparing cancer-specific survival (CSS) with pooled standardized mean difference for moderate 1 exercise group

**Fig. 2:**
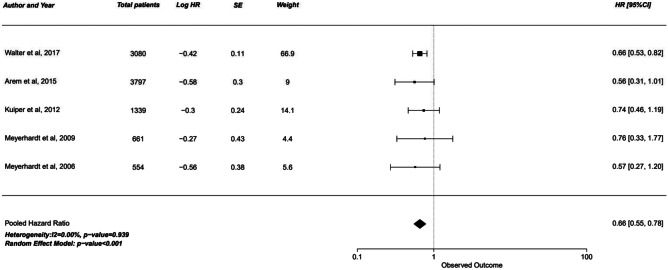
Forest plot of studies comparing cancer-specific survival (CSS) with pooled standardized mean difference for moderate 2 exercise group

**Fig. 3:**
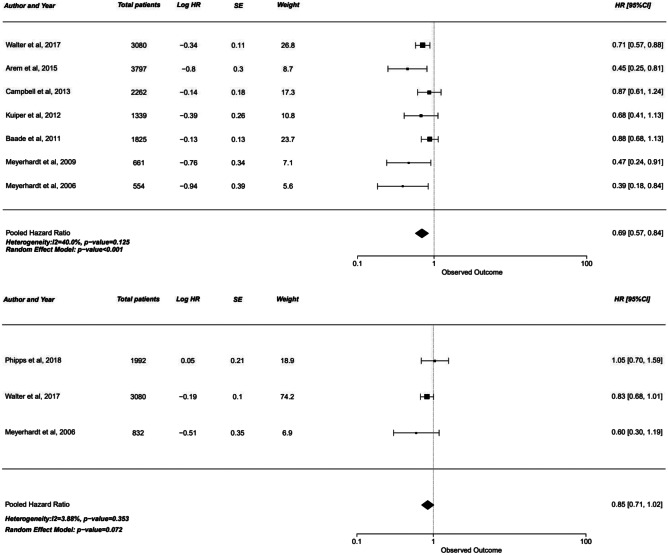
Forest plot of studies comparing cancer-specific survival (CSS) with pooled standardized mean difference for the highest intensity exercise group

In contrast, disease-free survival analysis showed no significant increase with physical activity. While the hazard ratio ranged from 0.85 to 0.92, all three groups had the upper limit of confidence interval crossing the 1.00 mark, negating the significance of this increase (Figs. 1, 2 and 3).

## Discussion

This updated meta-analysis of 19,135 patients with non-metastatic CRC once again shows that moderate physical activity per week is associated with a significantly decreased risk of overall mortality in CRC patients. Adding to current evidence, our sub-analysis found significant associations between physical activity and CRC-specific survival in non-metastatic CRC patients. However, the relationship between physical activity and disease-free survival, while previously suggested [20], deserves further investigation to determine significance — with only a small number of studies in our review (three) reporting on DFS.

Numerous biologic mechanisms have been suggested to explain the protective effect of physical activity on cancer mortality. For example, research in breast cancer patients show that apart from lowering blood pressure levels, exercise lowers the inflammatory marker C-reactive protein [20], suggesting an anti-inflammatory effect of exercise. On top of enabling a lower body mass index (BMI) [21], the resulting decrease in insulin levels and insulin-like growth with exercise has been hypothesized to have a role in improving overall immune function [21, 22]. Holistically, activity induced changes in the body and mental health also support improved tolerance for and the resultant effectiveness of cancer treatment [23]. These biologic mechanisms give a glimpse into the complex interplay between physical activity and cancer prognosis due to its effect on factors such as obesity, hormones, inflammatory cytokines and the immune system [24, 25]. Still very much a work in progress, we can conclude that further research on the biologic mechanisms involved in physical activity in CRC patients is highly warranted.

While optimizing a patient’s condition before surgery to improve postoperative outcomes remains the main aim of prehabilitation, long-term behavioural changes have been suggested as a secondary benefit [26]. Delivering this tailor-made “perioperative optimization package” at a time when patients is likely to be particularly amenable to behavioural change interventions could yield long-term gains [26, 27]. Lifestyle changes are complex but this preoperative period allows an opportune time to intervene in a multi-modal fashion — targeting lifestyle and physical activity levels [28, 29], diet and nutritional status in order to stave off deconditioning and sarcopenia [30], while enabling both short-term as well as long-term behavioural lifestyle changes and the resulting health benefits [31].

To this end, strategies on how to motivate patients and encourage longer term behavioural change deserve further study [27]. While some patients can be daunted by the seemingly gigantic undertaking of developing a healthy lifestyle upon diagnosis, they can be reassured by our results showing improved survival with moderate activity for example, walking alone [1]. Much has been said about the challenge of a relatively short period of 4–5 weeks between diagnosis and definitive surgery requiring close coordination between prehabilitation intervention programs and the treatment program [32]. The success was experienced in the CHALLENGE Trial, where locally implemented behaviour modification intervention translated into behavioural and health-related fitness improvements are especially noteworthy [33]. While the short-term outcomes reflect previous exercise interventions in cancer survivors [34], it shows promise that broader implementation will continue to produce small but significant behavioural and fitness changes [34]. If nothing, this highlights that empowerment of patients should not be underestimated, for it allows them to then play an active role in overcoming their disease [32].

Admittedly, our study has important limitations that deserve careful consideration. Although promising, the included studies are limited by the observational designs with high risk of confounding due to the use of self-report measures of physical activity. Ascertainment of physical activity was often done following a non-validated standardized baseline questionnaire. Thus, this assessment of physical activity was based on self-reported information, yielding potential for recall or other information bias. Furthermore, reported activities can vary between people in intensity or type, and misclassification can increase with increasing intensity of activity type. Patients could have generally overestimated their activity which might have led to an underestimation of associations between activity and prognosis.

Moreover, substantial study heterogeneity was found in several outcomes. This can limit the interpretability of the pooled estimates. Firstly, this study did not discriminate between pre and post diagnosis activity. Nonetheless, the studies included have a balanced representation of both groups. Additionally, individuals who are physically active before diagnosis often remain physically active during the post-diagnosis period as shown by positive correlations between pre-diagnosis physical activity and post-diagnosis physical activity amongst colorectal cancer survivors [33]. Pre-diagnosis physical activity may beneficially affect the treatment process because it leads to improved functional capacity to tolerate and complete surgery and adjuvant treatment [16].

Nonetheless, it is important to acknowledge that this heterogeneity also extended to possible differences in study population composition (e.g., age, smoking status) between the studies. Most but not all were explicit in reporting on the confounders listed above. This in turn could have translated to differences in the distribution of colorectal cancer attributes. While we have attempted to exclude a major confounder in the distribution of primary tumour sites, we still cannot exclude residual confounding by factors associated with physical activity, such as a healthier lifestyle, or lower prevalence of relevant comorbidities which might have led to an overestimation of associations between activity and improved survival.

Despite this, our review of 13 prospective studies looking at the impact of physical activity on colorectal cancer patients has shown physical activity to be associated with statistically and clinically important increase in overall survival and cancer specific survival. This is significant especially given the prevalence of colorectal cancer which ranks amongst the most common cancers both in men and women, especially in developed countries [1, 20]. Nonetheless, further randomized controlled trials are welcome to further assess the efficacy of physical activity on other health outcomes.
